# The Chicago Health and Life Experiences of Women Couples Study: Protocol for a Study of Stress, Hazardous Drinking, and Intimate Partner Aggression Among Sexual Minority Women and Their Partners

**DOI:** 10.2196/28080

**Published:** 2021-10-19

**Authors:** Cindy B Veldhuis, Lauren M Porsch, Lauren A Bochicchio, Jacquelyn Campbell, Timothy P Johnson, Allen J LeBlanc, Kenneth E Leonard, Melanie Wall, Sharon C Wilsnack, Mariah Xu, Tonda L Hughes

**Affiliations:** 1 School of Nursing Columbia University New York, NY United States; 2 School of Nursing Johns Hopkins University Baltimore, MD United States; 3 Department of Public Administration University of Illinois - Chicago Chicago, IL United States; 4 Health Equity Institute San Francisco State University San Francisco, CA United States; 5 Clinical and Research Institute on Addictions University at Buffalo Buffalo, NY United States; 6 Mailman School of Public Health Columbia University New York, NY United States; 7 School of Medicine & Health Sciences University of North Dakota Grand Forks, ND United States

**Keywords:** lesbian, bisexual women, intimate partner aggression, partner violence, same-sex couples

## Abstract

**Background:**

Large gaps exist in research on alcohol use and intimate partner aggression (IPA) among sexual minority women (SMW; eg, lesbian, bisexual). Dyadic research with SMW and their partners can illuminate how couple-level factors operate in conjunction with individual-level factors to shape well-being in this understudied and vulnerable population. Given the traditionally gendered lens with which women are primarily viewed as victims and men as perpetrators, understanding the dynamics of IPA in same-sex female couples can also advance research and practice related to IPA more generally.

**Objective:**

Guided by a recent extension of the minority stress model that includes relational (couple-level) sexual minority stress and the I-cubed theoretical perspective on IPA, we will collect individual and dyadic data to better characterize the links between hazardous drinking and IPA among SMW and their partners. First, this study aims to examine the associations among minority stress, hazardous drinking, and IPA in SMW and their partners. Minority stressors will be assessed as both individual and couple-level constructs, thus further extending the minority stress model. Second, we aim to examine potential mediators and moderators of the associations among minority stress, hazardous drinking, and IPA. Finally, we aim to test models guided by the I-cubed theoretical perspective that includes instigating (eg, relationship conflict), impelling (eg, negative affect and trait anger), and inhibiting (eg, relationship commitment and emotion regulation) or disinhibiting (eg, hazardous drinking) influences on IPA perpetration.

**Methods:**

This United States National Institutes of Health–funded project will draw from a large and diverse cohort of SMW currently enrolled in the Chicago Health and Life Experiences of Women (CHLEW) study—a 21-year longitudinal study of risk factors and consequences associated with SMW hazardous drinking. SMW currently enrolled in the CHLEW and their partners will be invited to participate in the CHLEW Couples Study. By analyzing dyadic data using actor-partner interdependence models, we will examine how each partner’s minority stress, hazardous drinking, and IPA experiences are associated with both her own and her partner’s minority stress, hazardous drinking, and IPA perpetration.

**Results:**

Data collection began in February 2021 and will likely continue through 2023. Initial results should be available by mid-2024.

**Conclusions:**

The CHLEW Couples Study will fill important gaps in knowledge and provide the basis for future research aimed at clarifying the causal pathways linking hazardous drinking and IPA among SMW. This will support the development of culturally appropriate targeted individual and dyadic prevention and intervention strategies.

**International Registered Report Identifier (IRRID):**

DERR1-10.2196/28080

## Introduction

### Background

Intimate partner aggression (IPA) is a serious public health problem that affects more than 1 in 3 women in the United States [1]. Although the definition of IPA continues to be debated [2], we consider IPA to include psychologically, physically, or sexually aggressive or coercive behaviors by a romantic or sexual partner [3]. Research on IPA among sexual minority women (SMW; eg, lesbian, bisexual) is relatively new; this research has primarily focused on heterosexual couples and largely, although not exclusively, on male-to-female aggression [4-6]. However, a growing body of literature suggests that SMW may be at an even greater risk of experiencing IPA than heterosexual women [7-12]. Among women surveyed in the Centers for Disease Control and Prevention’s 2010 National Intimate Partner and Sexual Violence Survey, 32% of heterosexual women, 42% of lesbian women, and 57% of bisexual women reported lifetime physical violence by an intimate partner [8]. Rates of psychological violence or aggression were also higher among lesbian (67%) and bisexual (73%) women than among heterosexual women (47%).

Ample research demonstrates strong linkages between hazardous drinking and IPA among heterosexual couples [5,6]. Hazardous drinking, defined by the World Health Organization as a pattern of alcohol use that increases the risk of harmful consequences (operationalized in this protocol using several indicators such as heavy drinking, heavy episodic drinking, and intoxication), is among the most prominent health-related disparities in comparisons of heterosexual women and SMW. Research examining the associations between IPA and hazardous drinking in SMW’s intimate relationships is limited [13]. Yet, in our research [14], and others’ [15-17], SMW report substantially higher rates of drinking alcohol, heavy drinking, and drinking-related problems than heterosexual women, which may increase the risk of IPA.

Drawing on the sample of SMW enrolled in the 21-year longitudinal Chicago Health and Life Experiences of Women (CHLEW) study, we will recruit the partners of our current participants to examine factors associated with hazardous drinking and IPA in this population. Specifically, we will take an innovative dyadic approach to test the influences of factors known to be associated with IPA perpetration and victimization among women generally (eg, hazardous drinking, relationship conflict, depression, and childhood abuse), as well as sexual-minority-specific factors (eg, sexual identity concealment and internalized stigma), along with new couple-level minority stress constructs that stem from society’s stigmatization of same-sex relationships.

### Alcohol Use and IPA Among SMW

Research in the general population has typically found a positive association between an individual’s drinking pattern and relationship dissatisfaction or IPA [18,19]. For example, a meta-analysis by Cafferky et al [18] indicated a statistically significant association between alcohol use, particularly problematic use, and both perpetration and victimization, replicating the previous 8 meta-analyses that examined alcohol use and IPA. Moreover, because of the expanded number of studies, this meta-analysis demonstrated that the association between alcohol use and perpetration was stronger for men than women, a finding that is consistent with earlier substantive reviews [19]. Although too few to permit meta-analysis, studies of couples’ drinking patterns from a dyadic framework have also demonstrated a relationship between alcohol use and relationship satisfaction or intimate partner violence. A number of large-sample longitudinal studies have demonstrated that the lowest satisfaction and the highest risk for divorce are found in couples in which one member is a heavy drinker, and the other is not [20,21]. The association between more nuanced measures of couples’ drinking patterns and intimate partner violence is somewhat more complicated, although it appears that heavy drinking by either member of the couple or by both is associated with an increased risk of IPA [22-24].

In contrast to studies among heterosexual couples, the few studies on alcohol use and IPA among SMW have largely been descriptive and have had multiple methodological limitations (eg, samples that overrepresent younger White participants or are too small or homogenous for subgroup analyses, lack of guiding theoretical frameworks or perspectives, and lack of dyadic research) [20,23,24]. Thus, the links between SMW's drinking and IPA are poorly understood. We know that hazardous drinking may contribute to or be an indicator of relationship stress and conflict in SMW’s relationships similar to heterosexual relationships. For example, Kurdek [25] asked same-sex couples to rate the issues about which they fought the most, such as finances, sex, and household tasks. Among same-sex female couples, the most frequent disagreements were about drinking or smoking [25]. Drabble and Trocki [15] found that SMW were almost 11 times as likely as heterosexual women to report relationship or social problems (eg, fighting and partner being angry) related to their drinking. Kelley et al [26] found that, controlling for psychological and physical aggression, lesbian women who reported discrepant alcohol use between themselves and their partners also reported poorer relationship quality.

A further limitation of research on the links between alcohol use and IPA among SMW is that it has typically focused on experiencing but not perpetrating IPA. For example, using data from the California Health Interview Survey, Goldberg and Meyer [27] found that both SMW and heterosexual women who binge drank on a daily or weekly basis had significantly higher odds of having experienced IPA. Research on the perpetration of IPA among SMW has tended to focus on discrepancies in drinking between partners as a potential causal factor. For example, in a longitudinal study of lesbian women, discrepant drinking was prospectively associated with being psychologically but not physically aggressive at 6- and 12-month follow-ups [28]. Being physically or psychologically aggressive at baseline was additionally associated with discrepant drinking. Thus, theoretically grounded research that includes both general and sexual minority–specific risk factors and that examines both IPA perpetration and victimization from the perspective of each partner is needed.

### Guiding Theoretical Perspectives

#### Minority Stress Theory

The predominant explanatory theory for health disparities among SMW is minority stress, which derives from the broader conceptualization of social stress as potentially harmful to health [29-31]. Sexual minority individuals are exposed to unique stressors on a continuum of proximity to the self. Most distal are objective stressors based primarily on the environment, such as discrimination and prejudice. These lead to more proximal appraisals of the environment as threatening, resulting in expectations of rejection or stigma. Most proximal are internalizations of negative social attitudes toward sexual minorities (internalized stigma) and the concealment of a sexual minority identity. As these stressors tax the ability to function on a day-to-day basis, they are associated with poorer psychological well-being and unhealthy coping behaviors [29,32-34], such as hazardous drinking [35] and possibly IPA [13,36].

In a systematic review of IPA and sexual minority–specific stressors, Longobardi and Badenes-Ribera [11] identified 10 studies of minority stress and same-sex IPA perpetration and victimization published between 2005 and 2015. The results indicated that internalized stigma, stigma consciousness, sexual identity concealment, and experiences of discrimination were each associated with both victimization and perpetration of IPA. However, the effect sizes for these associations were small to medium [37]. In a meta-analysis of risk factors for IPA perpetration and victimization, Kimmes et al [10] found internalized homophobia to be one of the strongest risk factors for IPA perpetration but not victimization among same-sex couples. Similar to research on other risk factors for same-sex IPA, nearly all studies of minority stress and IPA have focused on individual-level experiences. People in same-sex relationships, as well as those in other stigmatized relationship forms (eg, interracial or interethnic or intercultural couples), are exposed to both individual-level and couple-level minority stressors. For example, an SMW may hide her lesbian or bisexual identity from family members or friends who are perceived to be homophobic (individual-level minority stressor). However, when she is in a relationship with another woman, her status as a member of a sexual minority couple will result in exposure to additional stressors beyond those experienced at the individual level. For instance, she and her partner must jointly manage the visibility of their relationship and the possibility of rejection of them as a couple by families, religious communities, neighbors, and friends (couple-level minority stressor). This extension of the minority stress model to include couple-level stressors supports a more comprehensive examination of minority stress than its original conceptualization. A greater understanding of couple-level minority stress and its impact on hazardous drinking and IPA will provide important information that can inform couple-level interventions.

#### I-Cubed Model of IPA Perpetration

Decades of research has documented the impact of alcohol use on aggressive behavior. Generally focused on laboratory experiments of aggression between males, this literature has demonstrated that administering alcohol resulted in higher levels of aggression than administering no alcohol or a placebo [38] and that greater consumption of alcohol led to higher levels of aggression [39]. Explanations of alcohol’s effect on aggression centered on the cognitive disruption caused by intoxication; theorists argued that alcohol intoxication impairs an individual’s ability to attend to and process cues in a situation, resulting in *alcohol myopia*, in which behavior is more strongly affected by the dominant cues in the situation [40,41]. When cues are facilitative of aggression, alcohol consumption increases the likelihood of aggression; however, when cues are neutral or inhibitory, alcohol may, in fact, decrease the likelihood of aggression. In the context of aggressive cues, alcohol impairs cognitive functioning and reduces a person’s ability to self-regulate emotions and behavior. This, in turn, may impair the ability to restrain aggressive impulses. There is substantial support for this model of alcohol-related aggression [42].

**Figure 1:**
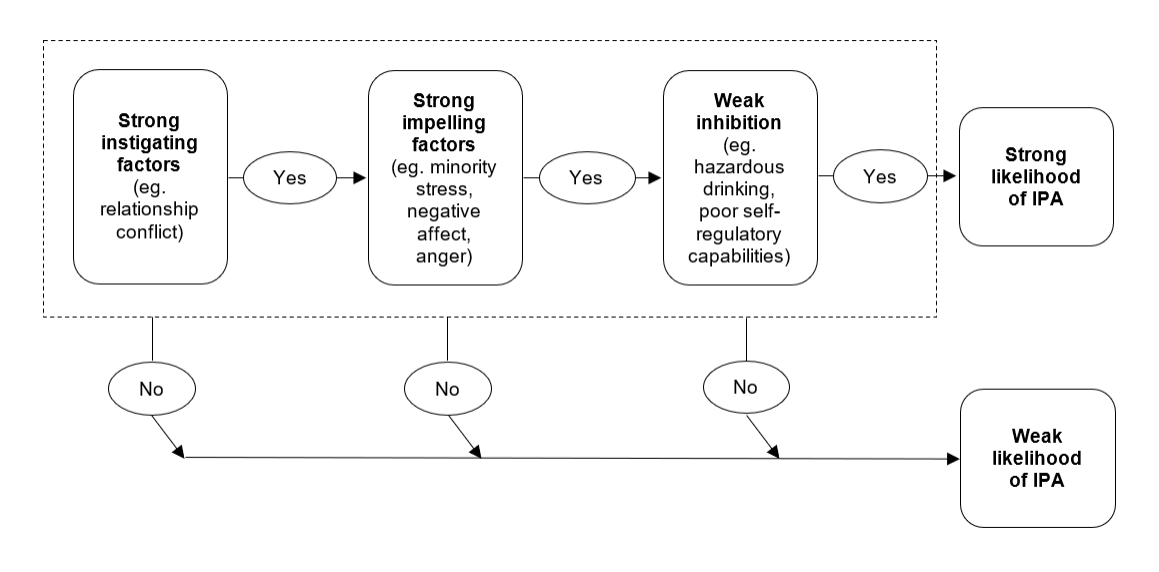
I-cubed theoretical perspective on intimate partner aggression perpetration among sexual minority women. IPA: intimate partner aggression.

To evaluate potential inhibitors or disinhibitors in the associations between hazardous drinking and IPA, we draw on the I-cubed (I^3^) model of IPA perpetration. This model incorporates the alcohol myopia theory into a larger framework for understanding the process by which a given factor promotes or mitigates aggression as well as how multiple factors interact to increase or decrease the aggression-promoting tendencies of the factor [43-45]. Results from experimental and longitudinal studies provide strong support for the I^3^ perspective and underscore the importance of self-regulatory processes in helping to reduce the risk of IPA. As illustrated in Figure 1, instigating triggers, such as relationship conflict, can set the stage for aggressive behaviors in a couple. For example, if the partner who experienced the instigating trigger also experiences high levels of minority stress or has a tendency to become angry easily (impelling factor), they will be more likely to respond aggressively. However, there are other factors that can act to inhibit aggression (eg, relationship commitment and emotion regulation) or act as disinhibitors (eg, alcohol consumption). We will test models that include both general factors stemming from the I^3^ framework and sexual minority–specific stressors (both individual and couple level) within a dyadic framework to understand how a diverse array of variables influence hazardous drinking and its association with IPA among SMW and their partners.

### Testing Dyadic Models Derived From Minority Stress and I3 Models

Research on IPA has historically taken a gendered perspective (women as victims or men as perpetrators), and for the most part this research has concentrated on either victims or perpetrators, not both simultaneously. Moreover, research on the links between hazardous drinking and IPA has rarely focused on the effects of both partners’ alcohol use. However, findings from existing studies (all with heterosexual couples) suggest that each partner’s alcohol use can independently predict both partners’ physical IPA perpetration. For example, Cunradi et al [46] found that among White and Black couples in which the female partner had alcohol-related problems, rates of female-to-male IPA were five to six times higher than in couples in which the female partner did not have alcohol problems. Among couples in which the male partner had alcohol problems, the risk of female-to-male IPA was 3 to 4 times higher than among couples in in which the male partner had no alcohol problems. These researchers found that among Hispanic couples, women’s alcohol problems were not associated with IPA perpetration. However, among Hispanic couples in whom the male partners had alcohol problems, the odds of IPA perpetration were more than 2 times higher than in couples in whom the male partner had no alcohol problems. Other research among heterosexual couples suggests that one partner’s heavy episodic drinking predicts the other partner’s anger (partner effects), and both actor and partner alcohol use predict physical and psychological IPA [47-49].

There is almost no dyadic research on hazardous drinking among sexual minority couples. In the only published study of which we are aware, LeBlanc et al [50] reported that in a sample of same-sex male and female couples, participants who felt that their relationships were not recognized to the same extent as the relationships of heterosexual couples (a couple-level minority stressor) had higher rates of problematic drinking. Moreover, results showed both actor and partner effects: one partner’s perceived unequal recognition was positively associated with the other’s problematic drinking [44].

Less well-understood are mediators and moderators of the hazardous drinking–IPA link, which is important for identifying modifiable mechanisms that may be used in prevention and intervention efforts. Although there has been research on mediators or moderators of the associations between alcohol use and violence, as noted above, much of it has focused on violence between men. Far less research is focused on couples. Research in this area tends to focus almost exclusively on factors that influence alcohol use and IPA in heterosexual couples [51]. For example, relationship dissatisfaction seems to play a mediating role in this link among heterosexual couples [51,52]. Among women in heterosexual couples reporting bidirectional violence, wanting to appear tough or wanting to intimidate one’s partner mediated the link between alcohol misuse and IPA. However, self-defense and the need to express negative emotions did not [53]. Whether the mediators and moderators of the associations between hazardous drinking and IPA are similar in SMW relationships is not yet known.

Although all SMW are exposed to minority stress, most do not perpetrate or experience IPA—even in the context of hazardous drinking—so there are clearly other factors that influence the risk of IPA in this population. Some evidence suggests that among women aggressors in heterosexual relationships, stress interacts with coping styles to increase aggressive behavior, and stress and coping may moderate the association between alcohol use and IPA [54]. Among lesbian women, general stressors have been found to be positively associated with hazardous drinking, and hazardous drinking is associated with IPA. Those who reported higher levels of emotional distress were more likely to drink to cope, consume more alcohol, and experience more drinking-related problems [55]. In a sample of lesbian women, Mason et al [56] found links between minority stress and physical IPA perpetration and that negative affect and interpersonal intrusiveness (eg, possessiveness) mediated this association.

**Figure 2:**
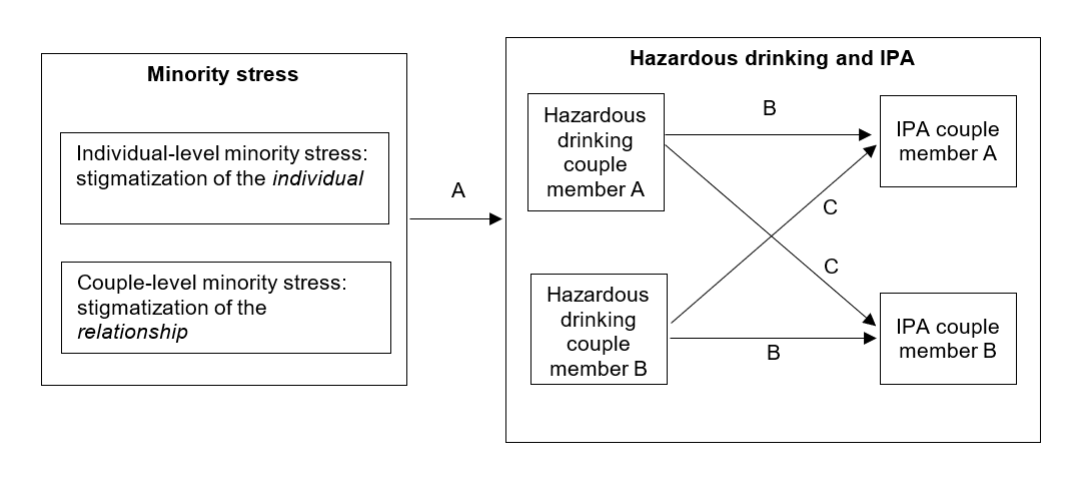
Extending the minority stress framework to include couple-level stressors. IPA: intimate partner aggression.

Together, these findings suggest that hazardous drinking may be an important contributor to IPA perpetration in SMW’s relationships, particularly among couples who experience high levels of minority stress. They also highlight the importance of examining alcohol-related IPA (inclusive of both partners’ reporting of perpetration and victimization) within a dyadic framework and potential mediators and moderators of these associations. For example, as illustrated in Figure 2, both individual- and couple-level minority stressors may influence the experiences of hazardous drinking and IPA perpetration among SMW and their partners (pathway A). The B pathways illustrate the hypothesized association between hazardous drinking and IPA perpetration for individual couple members (actor effects). The C pathways illustrate partner effects (the effects of each member’s hazardous drinking on their partner’s IPA perpetration).

### Aims

The specific aims of the study are discussed in the following sections.

#### Aim 1

Our first aim is to examine cross-sectional associations among minority stress, hazardous drinking, and IPA (perpetration and victimization) in SMW and their partners. We will test bivariate associations between hazardous drinking and IPA, and between individual- and couple-level minority stressors and hazardous drinking (heavy drinking, heavy episodic drinking, intoxication, and symptoms of potential alcohol use disorder) and IPA (physical, emotional or psychological, and sexual).

#### Aim 2

Our second aim is to examine potential mediators and moderators of the associations between minority stress and hazardous drinking and IPA (perpetration and victimization). Using dyadic actor-partner interdependence models (APIMs) and controlling for key variables, we will test potential mediators (eg, relationship conflict) and moderators (eg, relationship status and coping) of the associations between minority stress and hazardous drinking and IPA among SMW and their partners. We will also investigate whether the associations between hazardous drinking and IPA differ by sexual identity and race or ethnicity, as well as whether key psychosocial resources (eg, social support and positive coping) mediate the associations between minority stress and both hazardous drinking and IPA.

#### Aim 3

Guided by the I^3^ theoretical perspective, we will test models that include instigating factors (eg, relationship conflict), impelling factors (eg, minority stress, negative affect, and trait anger), and inhibiting (eg, relationship commitment and emotion regulation) or disinhibiting (eg, hazardous drinking) factors on IPA perpetration. Using dyadic data, we will examine a highly interactive model in which IPA perpetration is modeled as a function of both actor and partner minority stress, factors such as relationship conflict or commitment and hazardous drinking, and actor-partner interaction effects that take into account important areas of discordance (eg, differences in partners’ drinking patterns or experiences of minority stress) [22,57]. The models will control for key sociodemographic (eg, age, sexual identity, race or ethnicity, and length of relationship) variables.

## Methods

### Overview

In the baseline CHLEW study (wave 1; K01AA00266), we collected comprehensive data from a large and diverse sample of SMW who resided in the greater metropolitan Chicago (United States) area. This mostly descriptive study replicated and extended the National Study of Health and Life Experiences of Women (R01AA004610, SCW, PI), a 20-year longitudinal study of drinking patterns, problems, risk factors, and consequences among adult women in the general US population. Since wave 1, four follow-up waves of the CHLEW have been funded (R01AA013328-14, TLH, PI). Wave 2 extended the CHLEW to examine changes in alcohol use patterns and risk and protective factors for hazardous drinking, wave 3 examined the impact of accumulated childhood and adult stressors on drinking outcomes among SMW, and waves 4 and 5 (currently underway) focus on the impact of legalization of same-sex marriage in the United States on hazardous drinking and health. The procedures for each wave have been reviewed and approved by the institutional review board of the university where the principal investigator (TLH) held her primary appointment (waves 1-5 by the University of Illinois at Chicago; waves 4 and 5 and this study by Columbia University). Detailed information about the CHLEW study methods can be found elsewhere [58,59].

### CHLEW Couples Study Design and Sample

We will draw on the large cohort of SMW currently enrolled in the longitudinal CHLEW study for the recruitment of participants. Approximately half of the CHLEW cohort has been followed since 2000 and a half since 2010. We recruited the wave 1 baseline sample using social network or snowball sampling strategies with additional efforts to maximize sample representativeness. Women were eligible if they were aged ≥18 years, lived in the greater Chicagoland area, and self-identified as lesbian. Unlike most previous studies, participants (N=447) represented a wide age range (18-83 years), and more than half were women of color. In wave 2**,** we successfully located and reinterviewed 86% of the participants. In wave 3**,** we reinterviewed nearly 80% of all original participants (we were able to locate 85% of the women). In wave 3 (2010-2012), we also recruited a supplemental sample (N=373) of younger (aged 18-25 years), Black, Latina, and bisexual women.

As noted above, the sample was quite diverse in terms of race or ethnicity (35.9% Black, 23.1% Latinx, and 37.4% White). We will invite all CHLEW participants who currently have partners to participate in the CHLEW Couples Study. To determine eligibility, each CHLEW participant will be asked if they are dating or in a committed relationship of at least 3 months with an English-speaking partner aged ≥18 years. CHLEW participants will be asked to invite their eligible partners to participate with them in the CHLEW Couples Study. During the course of the CHLEW study, many participants have changed their sexual or gender identity; we will include otherwise eligible participants regardless of sexual or gender identity.

### Participant Recruitment and Retention

To aid retention in the longitudinal CHLEW parent study, participants have provided their social security numbers, cell phone numbers, and email addresses and listed the names, addresses, and phone numbers of 4 people who would always know their whereabouts. Letters with return postcards requesting address updates are sent to all participants at 6-month intervals. Other retention strategies included birthday and holiday cards, reminder calls before interview appointments, and graduated monetary incentives—from US $35 in wave 1 to US $80 in this study (US $40 for the telephone interview, US $20 for the web-based self-administered survey, and US $20 if the participant and their partner can be interviewed within 7 days of each other). Of the 820 participants enrolled in CHLEW (as of the writing of the grant application for this study), 49 (6.0%) were deceased, dropped out of the study, or were unable to participate for health reasons, leaving a sample size of 771 (94.0%) [59].

In previous CHLEW surveys, 61%-69% of participants reported that they were in a committed relationship; partial data from wave 4 (underway when the grant application was submitted) indicated that at least an additional 10% were in dating relationships. Although it is possible that the proportion of participants in relationships will be lower in the proposed study, we expect that it will be the same or possibly higher (given marriage equality and improved societal attitudes about sexual minority people and same-sex partnerships). Therefore, we estimate having a recruitment pool of at least 405 (71% of 771) CHLEW participants. In waves 4 and 5 of the longitudinal study, we asked all participants whether their partner (if they had one) was interested in participating in the CHLEW Couples Study. Again, using partially collected data from wave 4, we found that <10% said *no*. On the basis of a conservative estimate of 12% refusals, our sample would be 357 couples. However, CHLEW includes approximately 50 couples (100 women reported that their partner was also in the study), which reduces the estimated sample size to 307 couples. Of these, we expect that a few partners will not meet the eligibility criteria (age ≥18 years and able to speak English). Therefore, we budgeted for a total of 302 couples (604 individual interviews).

### Procedures

All SMW enrolled in the CHLEW parent study will receive an invitation from the principal investigator, describing the CHLEW Couples Study. Those in relationships of at least 3 months, whose partner is aged at least 18 years, and who can complete an interview in English will be invited to participate. The invitation letter will include a description of the study procedures, information about incentives (US $60 for each partner, plus an additional US $20 each if the couple agrees to be interviewed in the same week), and will emphasize confidentiality (eg, each member of the couple will have a different interviewer; no information from the interview will be shared with the participant’s partner or anyone outside the study team). In the invitation letters, CHLEW participants will be asked to call, text, or send an email message to the research office to indicate their interest. They will also be asked to have their partner contact the research office so that they can be screened and enrolled if eligible. If a partner does not contact the research office within 1 week, a member of the research staff will contact the index CHLEW participant to reassess the couple’s interest in participation. Participation will include a one-time, 60- to 90-minute telephone or videoconference interview conducted by a trained interviewer and a 25-minute web-based survey to be completed within 1 week of the telephone interview. We will ask each member of the couple to refrain from sharing information about the interview or survey until both partners complete the study.

We have collected data using interviews (face-to-face in waves 1 and 2 and telephone or Zoom in waves 3-5; we also completed approximately 100 wave 3 interviews by phone because many study participants had moved outside the Chicago Metropolitan area) as the primary mode of data collection. We feel that these interactions are key to our ability to retain such a high number of participants for 21 years. To assess potential mode effects in wave 3, we compared self-reports of alcohol and drug use among participants interviewed in person with those interviewed by telephone. Although women interviewed by telephone were less likely to report the use of cocaine, we found no differences in any of the hazardous drinking measures. These findings were consistent with the assessments of the 1990 and 2000 National Alcohol Surveys [60,61]. Our finding of limited mode effects in wave 3 provided confidence that this would not significantly influence the self-reports of key variables. Further, in wave 4, we asked participants how they preferred to be surveyed in the future (ie, phone, video, or on the web), and most people had no preference. We moved scales that are of interest but not central to the study’s aims to a web-based module to reduce possible participant fatigue associated with a long telephone or Zoom interview (Tables 1 and 2).

**Table 1:** Demographic questions.

Demographic	Number of items	Description of measure and method of administration	Role in study	Development and modifications
Sexual identity	1	Response options include only lesbian, mostly lesbian, bisexual, mostly heterosexual, only heterosexual, pansexual, queer, asexual, or none of the above. Participants are asked to specify a different term if “none of the above” is chosen. [Interview]	IV^a^ Covariate	Adapted from Skrocki [62]—revised to be inclusive of additional identities (eg, pansexual and asexual)
Sexual attraction	1	Response options include attracted to women, attracted to men, attracted to people with nonbinary identities, attracted to people of other genders, my attraction to people is not based on gender, not attracted to people of any gender, and not sure. Participants are asked to check all that apply and specify another term if “attracted to people with other genders” was selected. [Interview]	IV Covariate	Adapted from Skrocki [62]—revised to be inclusive of attractions other than to women or men
Sexual behavior	2	Past year sexual relationships with people other than the current partner (the partner who is also participating in the study). Response options include relationships with a woman or women, man or men, or nonbinary persons. [Interview]	IV Covariate	Adapted from Skrocki [62]—revised to be inclusive of relationships with nonbinary individuals
Sex or gender	1	We first ask about sex assigned at birth, then about current gender. Response options include female, male, transgender man or FTM^b^ transgender, nonbinary or genderqueer, and another gender identity. Participants are asked to specify a term if another gender identity is chosen. [Interview]	IV Covariate	Follows recommendations presented by Suen et al [63]
Race or ethnicity	2	We first ask about ethnicity (ie, Hispanic, Latina, Latinx, and Latino) and the second about race (response options include African American or Black, Asian or Pacific Islander, American Indian or Alaska Native, White, biracial or multiracial, and another race or ethnicity). If none of these apply, participants are asked to specify their racial or ethnic identities. [Interview]	IV Covariate	On the basis of questions asked in the 2010 census
Relationship status	1	In a committed relationship not living together, committed relationship living together, not in a committed relationship, or other; participants are asked to specify if “other” is chosen. [Interview]	Covariate	Adapted from Hughes et al [64]
Marital status	1	For participants in a committed relationship, response options include legally married, in a domestic partnership or civil union, or not married or in a domestic partnership or civil union. [Interview]	Covariate	From previous CHLEW^c^ waves
Education level	1	No formal schooling, eighth grade or less, some high school, high school diploma or GED^d^, some college or 2-year degree, bachelor’s degree, graduate or professional school [Interview]	Covariate	Adapted from Hughes et al [64]
Annual household income	1	“Looking at hand card #18, which of these groups represents your total annual household income from all sources? Household means everyone living in your house that you consider part of your family. Don’t include a roommate or housemate.” Response options include under US $1000 to US $9999 to >US $200,000 [Interview]	Covariate	From previous CHLEW waves
Managing finances as a couple	2	Two questions about how finances are managed as a couple; the first asks about each partners’ contribution to the total household income, and the second asks who decides how household income is used [Interview]	Covariate Moderator	Developed for the CHLEW Couples Study
Health insurance	2	Two questions about health insurance status; the first asks whether the participant has health insurance; a follow-up question asks if this is their own or their partner’s plan [Interview]	Covariate	Developed for the CHLEW Couples Study
Geographic location	1	In open country but not on a farm, on a farm, in a small city or town, in a medium-size city, in a suburb near a large city, or a large city [Interview]	Covariate	From previous CHLEW waves
Employment status	1	Working full-time for pay, working part-time for pay, unemployed and looking for work, managing the household, not looking for work, retired or disabled, not looking for work, for other reasons [Interview]	Covariate	From previous CHLEW waves
Number of children in the household	2	How many children younger than 18 years live in the household; then how many children older than 18 years live in the household. [on the web]	Covariate	From previous CHLEW waves

^a^IV: independent variable.

^b^FTM: female-to-male.

^c^CHLEW: Chicago Health and Life Experiences of Women.

^d^GED: General Educational Development.

**Table 2:** Description of major measures.

Scale			Number of items	Timeframe and method of administration	Role in study	Development and modifications
**IPA^a^**						
	CTS^b^; perpetration and victimization		22	Past 12 months [interview]	DV^c^	Adapted from Straus [65] Omitted verbal abuse items because of lack of sensitivity (verbal abuse is assessed using the Psychological Maltreatment of Women scale) Questions ask both about participant’s experiences of victimization and perpetration. Added questions after each section of the CTS about how often the participant and partner were each drinking
	Psychological Maltreatment of Women Inventory perpetration and victimization		30	Past 12 months [interview]	DV	Adapted from Tolman [66] Made pronouns gender neutral Added parallel questions about participants’ perpetration of psychological maltreatment Added questions about how often the participant and partner were each drinking at each IPA episode
**Alcohol use and hazardous drinking**						
	Drinking consequences		16	Past 12 months [interview]	Moderator	Adapted from Wilsnack et al [67] Modified from the NSHLEW^d^ and previous waves of CHLEW^e^; removed four questions that were not germane to the study aims
	Help-seeking for alcohol use		1	Past 12 months [interview]	Moderator	Adapted from Wilsnack et al [67]
**Hazardous drinking (participant’s own drinking)**						
	Heavy drinking		2	Past 30 days and past 12 months [interview]	Moderator or mediator	On the basis of guidelines from the NIAAA^f^ [68]
	HED^g^		2	Past 30 days and past 12 months [interview]	Moderator or mediator	On the basis of NIAAA guidelines and relevant research [67,69-71]
	Intoxication		1	Past 12 months [interview]	Moderator or mediator	On the basis of measures used in the NSHLEW [67] and in previous waves of the CHLEW; see also, Brunborg and Østhus [72]
	Maximum quantity drinking		1	Past 12 months [interview]	Moderator or mediator	From the national alcohol survey [61]
	AUD^h^		11	Past 12 months [interview]	Moderator or mediator	On the basis of DSM-5^i^ criteria for alcohol use disorder [73]
**Hazardous drinking (participant’s report of partner’s drinking)**						
	Heavy drinking		2	Past 30 days and past 12 months [interview]	Moderator or mediator	On the basis of guidelines from the NIAAA [68]
	HED		2	Past 30 days and past 12 months [interview]	Moderator or mediator	On the basis of NIAAA guidelines and relevant research [67,69-71]
**Smoking and other substance use**						
	**Smoking**					
		Cigarette smoking (yes or no)	1	Current [web-based]	Moderator	From previous CHLEW waves
		Change in smoking behavior from 12 months ago	1	Current [web-based]	Moderator	From previous CHLEW waves
		E-cigarette smoking (frequency)	1	Past 12 months [web-based]	Moderator	From previous CHLEW waves
		Vaping (yes or no)	1	Lifetime [web-based]	Moderator	From National Survey on Drug Use and Health [74]
	**Other substance use**					
		Substance use (sedatives, painkillers, marijuana, cocaine, stimulants, club drugs, hallucinogens, inhalants, heroin, or other; two questions about help-seeking)	12	Past 12 months [web-based]	Moderator or mediator	From the NESARC^j^ III [75] Replaced dichotomous yes or no responses with frequency scale consistent with the NESARC (never, monthly or less often, weekly, daily, or almost daily)
		DAST^k^-10	9	Past 12 months [web-based]	Moderator or mediator	Adapted from Skinner [76] and Yudko et al [77] DAST questions were asked if any of the NESARC items above were endorsed. The first item, which screens for any drug use, was redundant with the NESARC measures; it was omitted.
**Minority stressors**						
	Couple-level minority stressors		38	Life right now or past 12 months [interview and web-based]	IV^l^ Moderator or mediator	Adapted from Neilands et al [78]; used four subscales (of eight) that are most relevant to study aims: Couple-level expectations of rejection Couple-level discrimination Lack of integration with families of origin Lack of social support for couples
	Sexual identity disclosure		6	Current [interview]	IV Moderator or mediator	Adapted from Herek [79] and used in previous waves of CHLEW
	Discrimination scale		12	Past 12 months [interview]	IV Moderator or mediator	Items are based on the Experiences of Discrimination Scale. [80]. These were developed from the AUDADIS-IV^m^ study [81] and used in the NESARC-I [82]. Response options were amended to include additional reasons for discrimination; we ask separately about sex and gender.
	Family members’ reactions to disclosure		9	Lifetime [interview]	IV Moderator or mediator	Developed for CHLEW Study
	Internalized stigma		13	Current [web-based]	IV Moderator or mediator	Adapted from the Lesbian Internalized Homophobia Scale [83]
	Stigma consciousness		10	Current [interview]	IV Moderator or mediator	Adapted from Pinel [84] Items related to stigma related to being a lesbian Modified to create a bisexual version
**Mental and physical health**						
	Adverse childhood experiences (physical and sexual abuse and neglect)		8	Before age 18 [interview]	Moderator	From NESARC-III [85,86]
	Early Trauma Inventory		5	Before age 18 [interview]	Moderator	Adapted from Bremner et al [87] Psychological abuse subscale only
	Characteristics of childhood sexual abuse [88,89]		2	Before age 18 [interview]	Moderator	Adapted from Wyatt [88] and Wilsnack et al [89] and used in the NSHLEW and CHLEW
	Center for Epidemiological Studies Depression Scale		10	Past week [web-based]	Moderator or mediator	No modifications [90,91]
	Suicide ideation and attempts		3	Past year [interview]	Moderator	Adapted the diagnostic interview schedule: major depression [92]
	GAD^n^-7		7	Past 2 weeks [interview]	Moderator or mediator	No modifications [93]
	Self-rated physical health [94]		2	Past 30 days [interview]	Covariate	No modifications [94]
	Self-rated mental health [94]		1	Past 30 days [interview]	Covariate	No modifications [94]
	Therapy or treatment seeking		5	Past year [interview]	Moderator	Used in previous waves of CHLEW
**Impact of COVID-19**						
	COVID-19 diagnosis or symptoms		1	Since the beginning of the COVID-19 pandemic [interview]	Covariate Moderator	Developed for the CHLEW Couples Study
	Change to employment situation due to COVID-19		1	Since the beginning of the COVID-19 pandemic [interview]	Covariate Moderator or mediator	Developed for the CHLEW Couples Study
	Loss of health insurance due to COVID-19		1	Since the beginning of the COVID-19 pandemic [interview]	Covariate Moderator	Developed for the CHLEW Couples Study
	Change in alcohol consumption during COVID-19 pandemic		3	Since the beginning of the COVID-19 pandemic [web-based]	Covariate Moderator or mediator	Developed for the CHLEW Couples Study
**Relationship characteristics**						
	Couple satisfaction index		4	Current [interview]	Mediator	Adapted from Funk and Rogge [95] Removed the question about happiness
**Social support, stress, and coping**						
	Abbreviated brief COPE^o^ scale		26	Current or in general [web-based]	Mediator	Adapted from Carver [96] Removed venting items (items 9 and 21) Removed planning items (items 14 and 25) Removed humor items (items 18 and 28) Removed acceptance (items 20 and 24) Removed religion items (items 22 and 27)
	Brief resilience scale		6	In general [web-based]	Moderator or mediator	No modifications [97]
	Drinking Motives Questionnaire		5	In general [web-based]	Mediator	Adapted from Cooper [98] Coping subscale only
	LGB^p^ positive identity		5	In general [web-based]	IV Moderator or mediator	Adapted from Riggle et al [99] Community subscale only
	Multidimensional Scale of Perceived Social Support		12	Current [interview]	Moderator or mediator	Adapted from Zimet et al [100] Added follow-up asking whether responses to questions about family in the MSPSS^q^ referred to the participants’ family of origin, partner or children, or family of choice
	Perceived Stress Scale		4	Past month [web-based]	Moderator or mediator	No modifications [101]
**Anger, hostility, emotion regulation, negative affect**						
	BAQ^r^		12	In general [interview]	Mediator	No modifications [102,103]
	DERS^s^		12	In general [interview]	Mediator	Adapted from Kaufman et al [104] Removed two subscales (awareness and clarity)
	DAR^t^		5	In general [interview]	Mediator	No modifications [105]
	PANAS–SF^u^		10	In general [web-based]	Mediator	No modifications [106]

^a^IPA: intimate partner aggression.

^b^CTS: Conflict Tactics Scale.

^c^DV: dependent variable.

^d^NSHLEW: National Study of Health and Life Experiences of Women.

^e^CHLEW: Chicago Health and Life Experiences of Women.

^f^NIAAA: National Institute on Alcohol Abuse and Alcoholism.

^g^HED: heavy episodic drinking.

^h^AUD: alcohol use disorder.

^i^DSM-5: Diagnostic and Statistical Manual-5.

^j^NESARC: National Epidemiologic Survey of Alcohol and Related Conditions.

^k^DAST: Drug Abuse Screening Test.

^l^IV: independent variable.

^m^AUDADIS-IV: Alcohol Use Disorder and Associated Disabilities Interview Schedule-IV.

^n^GAD: generalized anxiety disorder.

^o^COPE: Coping Orientation to Problems Experienced Inventory.

^p^LGB: lesbian, gay, bisexual.

^q^MSPSS: Multidimensional Scale of Perceived Social Support.

^r^BAQ: Brief Aggression Questionnaire.

^s^DERS: Difficulties in Emotion Regulation Scale.

^t^DAR: dDimensions of Anger Reaction.

^u^PANAS–SF: Positive and Negative Affect Scale – Short Form.

### Study Instrument and Measures

The demographic measures for this study are included in Table 1. The baseline CHLEW instrument was adapted from the National Study of Health and Life Experiences of Women, which used measures with established reliability and validity. In addition to retaining hazardous drinking, sociodemographic, and other key measures from CHLEW’s previous surveys, we added measures that address new research questions and hypotheses relevant to the study aims, including measures of couple-level minority stressors. The CHLEW Couples Study survey instrument retained measures of major drinking variables, relationship variables (eg, relationship satisfaction, commitment, conflict, and IPA), as well as risk and protective factors for hazardous drinking (eg, depression, anxiety, resilience, and social support) included in the longitudinal CHLEW study. We also included several new measures. For example, we added questions about the frequency of physical, sexual, psychological, and verbal IPA—both victimization and perpetration—from the Conflict Tactics Scale [65] and the Psychological Maltreatment of Women Inventory [66]. Each of these measures has been used in prior studies that included SMW [20,107,108]. We also added new couple-level variables (eg, perceptions that the relationship is devalued) developed by LeBlanc et al [50,109]. To account for the potential effects of the COVID-19 pandemic, we included a few questions about the impact of the pandemic on participants, and we will include the date of the interview as a covariate to account for the timing of the interview and at what stage in the pandemic the interview occurred. Table 2 summarizes the measures of the major variables in this study.

### Data Analysis Plan

Most measures in the CHLEW Couples Study have established reliability and validity. Nevertheless, we will examine dimensional consistency and internal reliability of all scales and functioning across subgroups. We will correct outliers, data entry errors, or other logical inconsistencies. Given that we used a modified version of respondent-driven sampling to recruit the supplemental sample in CHLEW wave 3 [58], we will adjust for potential interdependence by including a sample cluster corresponding to the seed or referral chain (n=75 chains exist in our data) through which each supplemental sample participant was recruited. We will control for individual-level variables such as age, education, sexual identity, gender, race or ethnicity, education, date of interview, and other variables as appropriate in all APIMs. We may also control for couple-level variables such as relationship length, relationship status (eg, legally married), whether there are children aged <18 years living at home, and income. The choice of covariates will depend on the specific hypotheses being tested and will be guided by the current literature.

### Overview of APIMs

#### Overview

Analyses for each of the three aims will take full advantage of the dyadic data structure, where information is available on the same variables from each partner of the couple. Using APIM—a framework for analyzing interdependent dyadic data—IPA will be modeled as a function of both actor and partner experiences of their relationship and minority stress. APIM will also permit consideration of other potentially important factors influencing each partner’s IPA perpetration and victimization, such as actor and partner discrepant drinking patterns or discordant experiences of minority stress.

**Figure 3:**
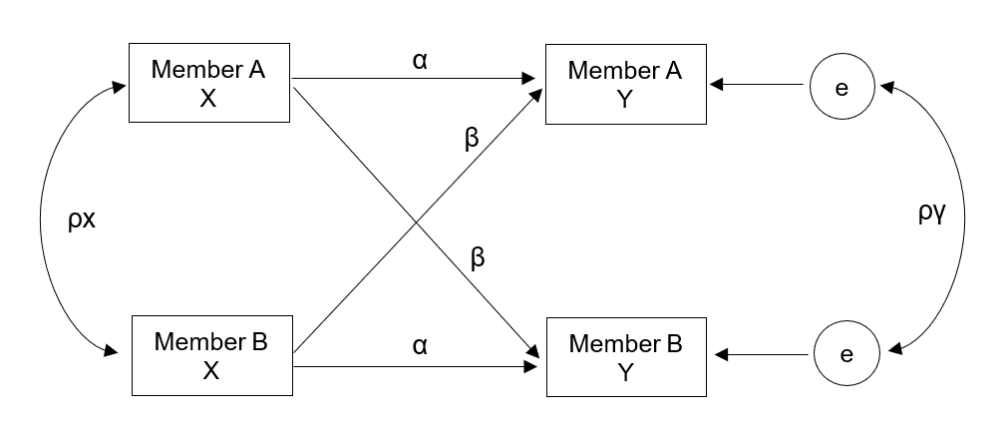
Basic actor-partner interdependence models for indistinguishable dyads.

We will implement APIMs at various levels of complexity to estimate the effects. As the sample will include predominantly same-sex partners, this results in the so-called indistinguishable dyads (who is labeled member A vs and who is labeled member B in the couple does not make a difference). All analyses will use APIM models constrained for indistinguishable dyads, regardless of whether the couple is same-sex or gender or mixed-sex or gender. That is, we will include all couples in the same indistinguishable dyad model (regardless of their sex or gender; we expect that <10% of couples will be mixed-sex or gender). Dyadic distinguishability (and the associated distinguishable model) refers to whether the two individuals within a dyad possess a distinctive characteristic that differentiates them in a manner relevant to the primary research question [110]. We will not elevate sex or gender as a distinguishable variable in the dyadic sense but instead, consider it a person-level covariate. Hence, we will use standard constraints within the APIMs such that actor effects will be fixed to be the same across participants, as will partner effects. In the basic APIM (Figure 3), α represents the extent to which the independent variable X of a participant influences their own score on the dependent variable Y (actor effect), and β represents the effect of the independent variable X of a participant on their partner's dependent variable Y (partner effect). In addition, we will obtain the intraclass correlation for the independent variable X, which is *p*x, and the intraclass correlation of Y after accounting for X, which is *p*y. Implementation of the APIM is available within the structural equation model framework in Mplus (Muthén and Muthén). We will control for individual-level variables such as age, education, sexual identity, race or ethnicity, length of relationship, and other variables as appropriate in all APIMs.

#### Aim 1

Although no studies have examined associations among minority stress, hazardous drinking, and IPA within a dyadic framework yet, we will examine a number of hypotheses derived from nondyadic research. We will use individual measures of minority stress as independent variables to predict each hazardous drinking outcome separately and then each IPA outcome (each partner’s reports of perpetration and victimization) separately. We will also fit models with each measure of hazardous drinking, predicting each measure of IPA. These effects represent the total (unmediated) effects of each variable on the other; they will show which measures are more or less strongly associated with one another, as well as which exhibit significant actor or partner effects. Additional models will test for differential effects by sexual identity, race or ethnicity, and longer- versus shorter-term relationships. These differential effects will be tested by including an interaction between X and each potential moderator in the APIM.

#### Aim 2

We will expand the APIMs tested in aim 1 to include multiple predictor variables, with some as mediators; we will also include interaction terms with potential moderating variables [111]. For example, to be a mediator of the relationship between minority stress and IPA, a variable M (eg, relationship satisfaction) must (1) exhibit a significant association with minority stress and (2) either show a significant association with the IPA outcome variable or, based on counterfactual theory, show a significant interactive effect with minority stress on the IPA outcome (each partner’s reports of perpetration and victimization). To test (1), we will fit the model in Figure 3 with the mediator as the outcome variable and test actor and partner effects (ie, the effect of minority stress on the mediator). Then, for (2), we will test the mediator—relationship satisfaction—as a predictor of IPA while also including minority stress in the model. We will use the bootstrap method to obtain standard errors and test the statistical significance of the mediation effect derived under the counterfactual framework, which allows for a possible interactive effect between relationship satisfaction and minority stress. We note that this inclusion of interactions in mediation effects goes beyond what is traditionally presented for mediation, for example, in Ledermann et al [111]; however, it is quickly being considered best practice and is implementable in Mplus [111-113]. Within the structural equation model framework, we can estimate these effects in a single integrated model, which allows us to estimate the mediation effect of actor or partner simultaneously. For example (*mediation hypothesis*): associations between minority stress (individual- and couple-level) and IPA will be mediated by each partner’s satisfaction with the relationship. Discrepancies in satisfaction will be associated with hazardous drinking in both partners, which in turn will be associated with IPA. Examples of *moderation hypotheses* are that hazardous drinking will moderate (ie, strengthen) the effects of minority stress and relationship dissatisfaction on IPA, and associations between minority stress (individual- or couple-level) and hazardous drinking or IPA will be moderated by each partner’s history of childhood abuse.

#### Aim 3

This aim is guided by the I^3^ theoretical perspective. We will address this using APIMs that include the effects of instigating factors (eg, relationship conflict), impelling factors (eg, minority stress, negative affect, and trait anger), and inhibiting factors (eg, relationship length or level of commitment and emotion regulation) or disinhibiting (eg, hazardous drinking) on IPA perpetration. We will operationalize IPA perpetration to be present for a member of the couple if either that participant reports perpetrating or their partner reports experiencing (ie, victimization) IPA. It is a well-established finding that intimate couples’ agreement on the occurrence of IPA is low to moderate [114]. Given social desirability concerns about reporting IPA, couple reports have been used in the large majority of dyadic IPA studies (based on the assumption that couple members are more likely to deny an actual occurrence than to falsely report IPA). Conceptually, the I^3^ perspective requires certain combinations of variables within both the actor and partner and across the 3 domains (instigating, impelling, and inhibiting) to be present if the risk of IPA perpetration is strong. Hence, we will include interactive effects (cross-products) between actor and partner predictor variables (eg, partner A’s hazardous drinking with partner B’s negative affect) within the APIM. The model will also include actor and partner interactions on the same variables corresponding to concordance (both partners drink heavily, neither partner drinks heavily, etc) and discordance. Tests of the significance of these different interactions will provide the necessary information for each step of the I^3^ model. An *example hypothesis* is that partner A’s hazardous drinking and partner B’s negative affect will interact to predict partner A’s IPA perpetration.

Given our planned sample size of 302 dyads (604 individuals), with two-sided tests and α=.05, we will have >80% power to detect small associations (ie, standardized regression coefficients as small as 0.12) in the full sample. Although not central to the study aims described above, we plan to examine demographic differences in the associations between hazardous drinking and IPA. On the basis of the racial/ethnic and sexual identities of CHLEW participants and assuming that most partners will have similar characteristics, our sample will include approximately 221 Black and 143 Latinx SMW and 206 bisexual women. The anticipated sample size will provide good power (>80%) to detect small-sized associations (ie, standardized regression coefficients as small as 0.20 in bisexual and Black and 0.23 in Latinx participants) in each subgroup. These detectible effect sizes apply to all the direct effect estimates of interest in aims 1, 2, and 3 [115,116]. The power to detect interactive effects is driven primarily by the sample size in the smallest cells of the interaction. We also have acceptable power to detect such interactions. For example, if the effect size of minority stress on heavy drinking for lesbian women is 0.20 and the effect for bisexual women is 0.40, we will be able to detect with 80% power that these effects are different (ie, significant minority stress × sexual identity interaction). Moreover, bisexual women and SMW of color are more likely than lesbian and non-Hispanic White women to report heavy drinking or drinking-related problems and IPA, increasing the likelihood of detecting significant race/ethnicity by sexual identity interactions [7,8,16,117-120]. On successful completion, this study will be the largest couple cohort study of its kind. The large sample size and large subgroup sizes will provide good power to detect clinically and practically meaningful effects.

## Results

Data collection for this project began in February 2021 and will continue through 2023. Preliminary data are expected to be available in mid-2024.

## Discussion

### Principal Findings

The changing social landscape, including the legalization of same-sex marriage, has led to heightened interest in research on same-sex relationships; however, this topic is substantially underrepresented in the literature. Furthermore, although research on alcohol use and IPA among sexual minority people has grown in the past 2 to 3 decades, important gaps remain, which the CHLEW Couples Study will address. First, most research on SMW has focused on the prevalence of IPA rather than on individual, relational, and contextual factors. Second, existing studies have focused predominantly on the main effect of associations between hazardous drinking and IPA. Less examined and understood are potential mediators and moderators of these associations, which are particularly important in informing interventions. Third, there are very few published studies on the association between hazardous drinking and IPA among SMW that have included both partners, and even fewer have examined both victimization and perpetration. Addressing these gaps represents an important shift from our (and others’) predominant focus on individual risk factors to also considering couple-level factors. Finally, sample sizes of sexual minority people in population-based studies are typically small, and subsamples of those who are both sexual and racial or ethnic minorities are even smaller; consequently, research on subgroups (eg, racial or ethnic minority SMW) is very rare.

This study is guided by minority stress and IPA theoretical perspectives to better understand both individual and dyadic factors that promote or deter hazardous drinking and IPA among SMW and their intimate partners. Building on our previous work that examined the impact of hazardous drinking on general and sexual minority–specific stressors that accumulate throughout the life span, we will capitalize on an opportunity to examine the links among minority stress, hazardous drinking, and IPA. This study also has a more balanced perspective that considers both risk and resilience from the perspectives of both members of the couple. We hope to advance the fields of sexual minority health and women’s health. Specifically, we will add significantly to the currently sparse knowledge about individual, interpersonal, relational, and contextual factors that contribute to elevated rates of physical and mental health problems among SMW.

### Limitations and How They Will Be Addressed

In addition to the strengths outlined above, we recognize the limitations of the study. First, it is possible that some study participants may be reluctant to share information about IPA in their relationship because of feared loss of confidentiality or social desirability. These fears may be complicated by the COVID-19 pandemic, given that couples who live together may have less privacy because of shelter-at-home guidelines. For purposes of confidentiality, partners will be interviewed by separate interviewers and assured that no information they provide will be shared with their partners and that all data will be treated as confidential. We will provide all participants with earbuds (with microphones) to help protect privacy (ie, so that others in the household cannot hear the questions being asked of the participant). We will employ interviewers experienced in conducting interviews that ask questions about sensitive topics and will provide them with extensive training on how to ensure participant privacy and maintain confidentiality. To reduce social desirability bias, we will preface IPA questions with a brief introduction that contains additional confidentiality assurances [121-123]. Also, to address the possibility of underreporting of IPA by one or both members of a couple, we will compare their reports of IPA perpetration and victimization. We will consider IPA to have occurred if either partner reports having perpetrated or experienced a particular form of IPA. We will conduct additional analyses of cases in which partners provide discrepant reports of perpetration or experience of IPA to gain additional insights about each partner’s experiences. We will also examine variables such as hazardous drinking, that may be associated with discrepancies in partner perceptions of IPA.

Second, we expect to have a small number of partners who are men or report transgender or nonbinary gender identity. We will conduct exploratory analyses to glean information about potential similarities and differences in findings based on the sex or gender of partners. As we will not know how many mixed-gender dyads we will have in our final sample until the recruitment is over, it is unclear whether we will have large enough subsample sizes of, for example, transgender partners or cisgender men partners to examine couple-level gender differences. There is very little research on couples among whom at least one member is transgender; however, there is some suggestion in the literature that alcohol use may be influenced by unique aspects of those relationships [124-126]. There is also little research with SMW in relationships with cisgender men; however, available data suggest that partner gender plays a key role in drinking-related behaviors in these couples [127-129].

Third, our past experiences of scheduling interviews with individual CHLEW participants suggest that it will take more time and effort to schedule both the CHLEW participant and their partner’s interview within close proximity (the same week). As it is important that the same periods be reflected in reports from each member of the couple, we will provide a US $20 incentive to each member of the couple who is willing to be interviewed in the same week (in separate, private locations). As we will train at least 5 interviewers and because research staff will also conduct interviews, we can be flexible and accommodate most schedule requests. Finally, the CHLEW Couples Study uses a cross-sectional design. For this reason, inferences about causal relationships will be considered cautiously, with careful attention to assumptions about causal ordering that cannot be directly tested.

### Implications for Intervention

We expect that the findings of this study will provide the basis for future research aimed at clarifying the causal pathways linking hazardous drinking and IPA among SMW. This research can support the development of individual and dyadic prevention and intervention strategies for SMW and their partners. Research with heterosexual couples suggests that relationship factors are especially important to consider in intervention development [130-132]. Given the existing empirical evidence, there is reason to believe that dyadic interventions are particularly well-suited for treating IPA, especially among couples who do not wish to separate. We are particularly interested in gaining information about modifiable targets for intervention—both general (eg, couple communication, alcohol use behaviors, and emotion regulation) and sexual minority–specific (eg, conflicts about differing levels of sexual identity disclosure)—that can be used in working with SMW. For example, if we find that rejection from families of origin is a particularly strong modifier of the link between minority stress and IPA among couples in the study, dyadic interventions that aim to help couples cope with familial rejection and find other sources of support could be tested.

## Abbreviations

APIM: actor-partner interdependence models
CHLEW: Chicago Health and Life Experiences of Women
I^3^: I-cubed
IPA: intimate partner aggression
NIAAA: National Institute on Alcohol Abuse and Alcoholism
NIH: National Institutes of Health
SMW: sexual minority women
